# Goodbye to the paper journal and welcome to our new open access format

**DOI:** 10.1093/eurpub/ckab216

**Published:** 2022-02-01

**Authors:** Peter Allebeck, Dineke Zeegers Paget

**Affiliations:** 1 Department of Global Public Health, Karolinska Institutet, Stockholm, Sweden; 2 European Public Health Association, Utrecht, The Netherlands

With this issue, we implement the new format of the *EJPH*, improving accessibility to the journal, as well as abandoning the hard print copies of the *EJPH*. 

The change to full Open Access was decided by the European Public Health Association in November 2020. With our switch to full Open Access, not only our members but also all public health professionals have access to the publications of high-quality scientific research. On top of that, we also fully comply with the European Union’s demand for Open Access for all research.

With the new online-only version of the *EJPH*, we say goodbye to the hard copy of the journal. And this issue is the first that will not be distributed by regular mail to our subscribers and to libraries in Europe and in the world. While some of us like paper journals, to carry with us for reading on buses and trains, etc., this is not the way scientific articles are read today. The latest report from the publisher shows that readers access papers in the journals for more than 50% through Google or Google Scholar, around 25% access articles directly on the web, and only few through other channels. In the interest of the environment and bookshelf space in our offices (now to a large extent at home), we do not think one should be sad.

While our change in format is an adaptation to how scientific papers are read and shared, we have been very keen on not changing the content and structure of our journal. Many open access journals are not really journals, but catalogues of papers, sometimes sorted in some way, often not. We believe that a journal’s ‘soul’ is reflected not only by its scientific content but also by its additional material, such as editorials, viewpoints and our ‘European public health news’. While not clearly visible in statistics, many browse journals’ tables of content for assessing where to publish articles or finding linked articles in thematic issues.

We welcome readers and authors to our new format of the journal, more adapted to current ways of accessing articles, and in line with the general move to open access to scientific information. You will not get the journal in your postbox, but the content and structure of the journal will remain unchanged.

*Conflicts of interest*: None declared. 

